# Neutrophil Elastase (ELANE) as a Novel Neuroinflammatory Biomarker in Alcohol Use Disorder: Clinical Validation

**DOI:** 10.1111/adb.70103

**Published:** 2025-12-10

**Authors:** Bo Zhang, JinLing Zhang, MengYa Zhu, Yong Fan, Yong Xue

**Affiliations:** ^1^ Department of Psychology Shandong Provincial Hospital Affiliated to Shandong First Medical University Jinan Shandong China; ^2^ Qingdao Central Hospital University of Health and Rehabilitation Sciences (Qingdao Central Hospital) Qingdao China; ^3^ The Third People's Hospital of Huai'an Huai'an China; ^4^ Department of Psychiatry Qingdao Mental Health Center Qingdao Shandong China

**Keywords:** alcohol use disorder, ELANE, relapse biomarkers

## Abstract

Alcohol use disorder is a severe public health problem; however, the specific mechanisms remain unclear. Our previous studies have identified ‘ELANE’ as the Hub gene in alcohol use disorder. However, its role in the clinical practice of alcohol use disorders has not been confirmed. A total of 53 healthy controls and 90 patients with alcohol use disorders were enrolled. Clinical factors were gathered, and a 1‐year relapse follow‐up was carried out. The group with alcohol use disorder had considerably higher ELANE concentrations than the healthy controls (*p* < 0.001). Patients were categorized as high ELANE (≥ 2.7651 pg/mL, *n* = 46) or low ELANE (< 2.7651 pg/mL, *n* = 44) based on the median ELANE expression level in the alcohol use disorder group. SERPINA3 was statistically significant, according to binary logistic analysis (*p* = 0.007). After 12 months of follow‐up, there was no difference in event‐free survival between patients with low and high ELANE levels, according to Kaplan–Meier survival analysis (*p* = 0.568). ELANE had an area under the curve of 0.8683 (*p* < 0.0001), according to receiver characteristic curve analysis, and a sensitivity and specificity of 65.6% and 92.5%, respectively. According to Cox regression analysis, marital status was a negative predictor of relapse (β = −0.661; hazard ratio = 0.516; *p* = 0.038). Plasma ELANE represents a promising neuroinflammatory biomarker for AUD diagnosis, demonstrating excellent specificity albeit moderate sensitivity. The protease–antiprotease imbalance reflected by elevated ELANE and relatively decreased SERPINA3 suggests dysregulated inflammatory homeostasis in AUD. While ELANE lacks prognostic utility for relapse prediction, these findings warrant further investigation of neutrophil elastase inhibitors as potential therapeutic interventions and highlight the critical importance of social support systems in AUD recovery.

## Graphical abstract

Plasma neutrophil elastase(ELANE) was markedly elevated in patients with alcohol use disorder, indicating a dysregulated protease‐antiprotease inflammatory balance characterised by decreased SERPINA3. ELANE showed excellent diagnostic specificity(AUC = 0.8683), supporting its potential utility as a neuroinflammatory biomarker for AUD. However, ELANE did not predict relapse, emphasising the importance of social and clinical factors in long‐term recovery.

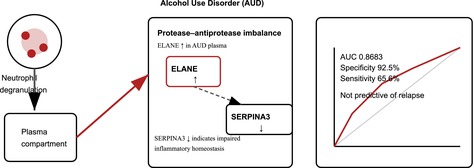

## 1 Introduction

Alcohol use disorder (AUD) is a highly prevalent chronic condition imposing a profound social and medical burden. Etiologically, AUD risk is estimated to be approximately 50% genetic and 50% environmental [1]. Neurobiology, epigenetic adaptation [2] and neuroimmunity [3] may play a role in the development of AUD, but further research is needed. Contemporary neuroscientific research has increasingly implicated neuroinflammatory cascades, neuroimmune dysregulation and blood–brain barrier dysfunction as central mechanisms underlying AUD development, maintenance and relapse vulnerability [3]. The neuroinflammation hypothesis posits that chronic alcohol exposure initiates a self‐sustaining cycle of inflammation, driving continued consumption and negative affective states associated with withdrawal [4]. Landmark research in 2024 definitively established that alcohol consumption induces neuroinflammatory responses and BBB injury via the purinergic P2X7 receptor signalling pathway [5]. Specifically, ethanol‐induced mitochondrial damage leads to increased adenosine triphosphate (ATP) release, P2X7 receptor activation and subsequent upregulation of proinflammatory gene expression profiles in brain microvessels [5]. This inflammatory milieu, involving microglial activation, astrocytic dysfunction and peripheral immune cell infiltration [6], disrupts synaptic homeostasis, impairs neuroplasticity and contributes to the characteristic cognitive and emotional deficits of AUD [7]. Neuroinflammation is thus a pivotal mechanism in AUD neurobiology [8, 9]. AUD may act as a pro‐inflammatory stimulus, inducing a neuroinflammatory response and promoting stress‐induced craving through the regulation of neuroimmune movements by microglia and astrocytes [6].

Our previous research has identified ‘ELANE’ as a key gene for alcohol use disorder [10]. ELANE encodes neutrophil elastase (NE), a 30‐kDa serine protease predominantly stored in azurophilic granules and released during neutrophil degranulation [11]. Beyond its established antimicrobial functions, NE exhibits pleiotropic biological activities including extracellular matrix degradation and inflammatory signalling modulation [12, 13]. Mechanistically, NE activates TLR4 and EGFR to activate the inflammatory response [12, 13]. Furthermore, NE inhibits innate immune function by inhibiting macrophage phagocytosis [14]. The role of neutrophil elastase in neuroinflammatory diseases is increasingly recognized, and emerging research suggests that it may serve as both a biomarker and a therapeutic target [15]. Collectively, these findings suggest that neutrophil elastase (ELANE) functions as a critical mediator in the neuroinflammatory process. Given the central role of inflammation in alcohol‐related neurotoxicity, ELANE represents a biologically plausible and previously underexplored biomarker candidate for alcohol use disorder. Investigating its plasma levels in patients with AUD may therefore provide novel insights into the peripheral inflammatory mechanisms underlying disease pathogenesis.

The biological activity of neutrophil elastase is tightly regulated by endogenous serine protease inhibitors, particularly SERPINA3 (serpin family A member 3), also known as α1‐antichymotrypsin [16]. SERPINA3 represents an acute‐phase inflammatory glycoprotein that functions as the primary circulating inhibitor of neutrophil‐derived serine proteases [17]. Recent transcriptomic analyses across multiple psychiatric disorders have consistently identified SERPINA3 dysregulation in brain tissue, cerebrospinal fluid and plasma, suggesting its involvement in neuroinflammatory processes [18]. However, controversy persists regarding whether SERPINA3 exerts predominantly protective or pathogenic effects in central nervous system disorders [19].

This study was conducted to investigate the potential role of the ELANE gene in alcohol use disorder, which may be a potential biomarker or therapeutic target for alcohol use disorder.

## 2 Methods

### 2.1 Clinical Samples

All participants' baseline laboratory and clinical indicators were collected. AUD diagnosis was confirmed using the *Diagnostic and Statistical Manual of Mental Disorders‐Fifth Edition* (DSM‐V) criteria. All enrolled subjects were between 18 and 65 years old and underwent semi‐structured clinical interviews conducted by trained psychiatrists. All participants were assessed through semi‐structured clinical interviews, administered by trained psychiatrists. Exclusion criteria included current or past diagnosis of other substance use disorders (except nicotine dependence), major psychiatric or neurological disorders severe medical conditions. All patients were hospitalized for detoxification and had maintained at least 24 h of abstinence before blood sampling. Healthy controls had no history of psychiatric or substance use disorders and were matched for age and sex. All participants provided written informed consent. This study was authorized and approved by the Ethics Committee of the Third People's Hospital of Huai'an City.

### 2.2 Sample Collection and Processing

All blood samples were collected from fasting participants between 7:00 a.m. and 9:00 a.m. and centrifuged at 3000 *g* at room temperature for 15 min. The plasma supernatant was removed and stored at −80°C. ELISA kit (ELANE Human ELISA Kit, YanZun, Shanghai, China) was used to detect ELANE concentration in each plasma sample according to the manufacturer's protocol. Each plasma sample was analysed in triplicate, and the mean value was used for statistical analysis, ensuring high reproducibility and assay reliability.

### 2.3 Longitudinal Follow‐Up and Relapse Assessment

Participants underwent systematic follow‐up assessments at 1, 3, 6, 9 and 12 months post‐discharge. Family members or designated contacts provided collateral information when permitted by participants.

## 3 Statistics

Multiple linear regression analysis was used to determine the variables most closely related to ELANE level. The receiver operating characteristic (ROC) curve and area under the curve (AUC) were analysed to test the diagnostic value of ELANE for alcohol use disorder. Kaplan–Meier survival curve was used to compare the recurrence time to ELANE level between the high ELANE group and the low ELANE group. Cox regression analysis was used to determine the risk factors for relapse.

## 4 Results

### 4.1 Experimental Validation of ELANE

**FIGURE 1:**
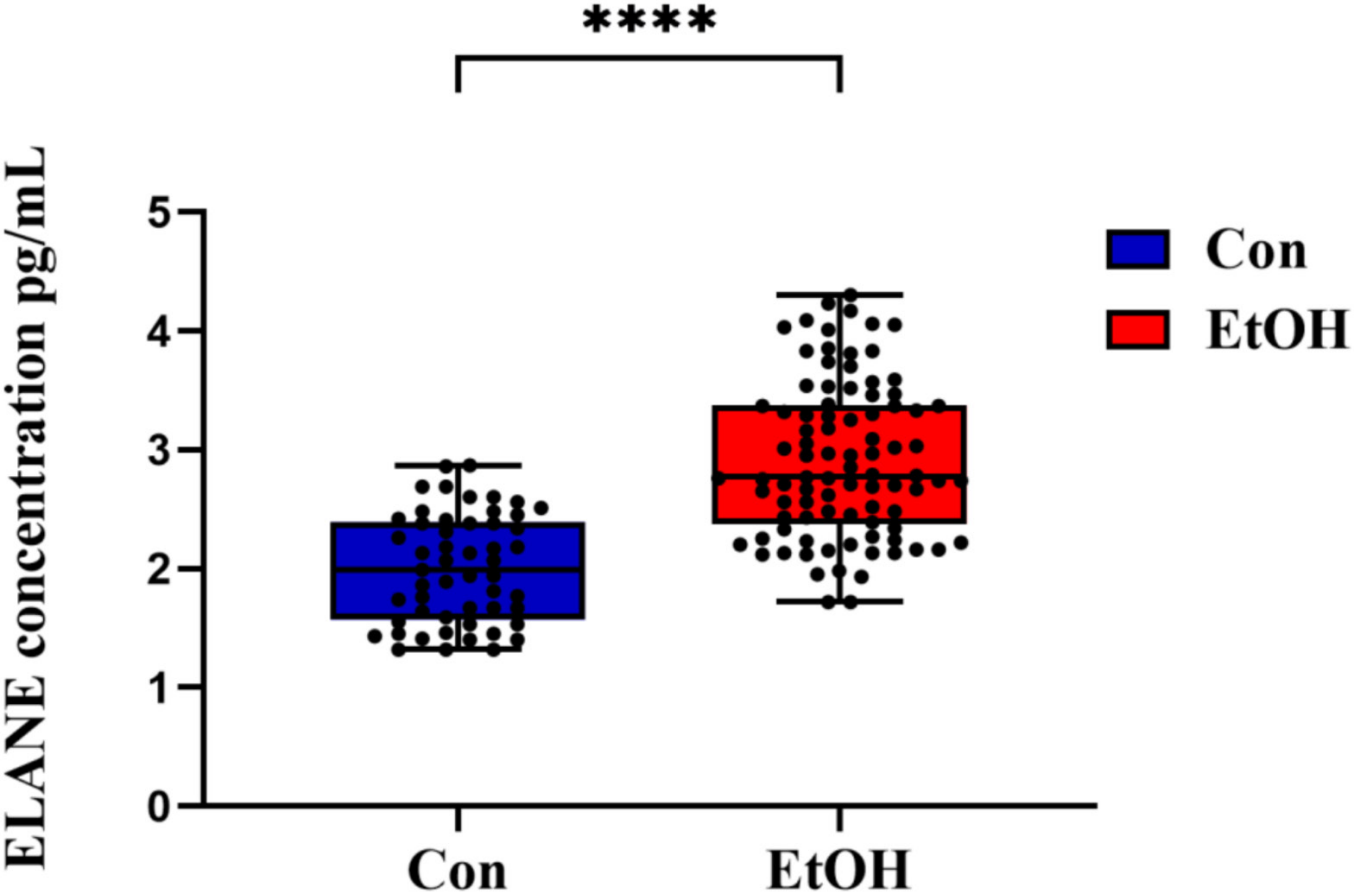
Comparison of plasma ELANE levels between patients with alcohol use disorder and the healthy control group.

Table 1 presents the comparison of general characteristics between the alcohol use disorder (AUD) group and the healthy control group. The results show that there were no statistically significant differences between the two groups in terms of gender, age or marital status. ELISA was used to verify that the concentration of ELANE in patients with alcohol use disorder was significantly higher than that in healthy controls (*p* < 0.001) (Figure 1). Patients were divided into a high ELANE group (≥ 2.7651 pg/mL, *n* = 46) and a low ELANE group (< 2.7651 pg/mL, *n* = 44) based on the median ELANE level. Table 2 compares clinical trial data between the two groups. The results show that the higher the concentration of ELANE, the lower the concentration of SERPINA3, showing a negative correlation (*p* = 0.004). Further binary logic analysis showed that the SERPINA3 difference was statistically significant (*p* = 0.007) (Table 3).

**TABLE 1:** Comparison of general characteristics between the alcohol use disorder (AUD) group and the healthy control group.

	Alcohol use disorder (*n* = 90)	Health control (*n* = 53)	*p*
Demographics			
Age, years	45 ± 11	41 ± 12	0.155
Gender			0.890
Male, *n* (%)	87 (96.7%)	51 (96.2%)	
Female, *n* (%)	3 (3.3%)	2 (3.8%)	
Marital status, *n* (%)			0.066
Unmarried	10 (11.1%)	8 (15.1%)	
Married	65 (72.2%)	43 (81.1%)	
Divorced	15 (16.7%)	2 (3.8%)	

**TABLE 2:** Correlation analysis between high and low concentration ELANE and laboratory indexes.

	High ELANE level (*n* = 46)	Low ELANE level (*n* = 44)	*p*
Demographics			
Age, years	46 ± 10	47 ± 11	0.722
Gender			0.584
Male, *n* (%)	44 (95.7%)	43 (97.7%)	
Female, *n* (%)	2 (4.3%)	1 (2.3%)	
Education (year)			0.215
≤ 6	14 (63.6%)	8 (36.4%)	
6–12	19 (42.2%)	26 (57.8%)	
≥ 12	13 (56.5%)	10 (43.5%)	
Marital status, *n* (%)			0.668
Unmarried	4 (40%)	6 (60%)	
Married	35 (53.8%)	30 (46.2%)	
Divorced	7 (46.7%)	8 (53.3%)	
Occupation, *n* (%)			0.530
Mental labour	6 (66.7%)	3 (33.3%)	
Physical labour	15 (53.6%)	13 (46.4%)	
Unemployed	25 (47.2%)	28 (52.8%)	
BMI, kg/m^2^	23.08 ± 3.70	22.61 ± 3.47	0.537
Drinking duration (year)	20 ± 10	20 ± 10	0.952
Heart rate, bpm	94.98 ± 14.86	98.67 ± 15.44	0.257
Systolic blood pressure, mmHg	134 ± 17	138 ± 18	0.276
Diastolic blood pressure, mmHg	88 ± 10	910 ± 12	0.451
Current smoker, *n* (%)	37 (48.1%)	40 (51.9%)	0.158
Laboratory tests			
RBC	6.44 ± 14.78	4.25 ± 0.56	0.329
Haemoglobin, g/L	138.93 ± 24.65	140.66 ± 15.60	0.692
MCV	100.60 ± 7.98	99.74 ± 7.87	0.607
Leukocytes, ×10^9^/L	7.07 ± 2.54	7.22 ± 2.37	0.770
Neutrophil, ×10^9^/L	4.99 ± 2.40	5.01 ± 2.25	0.975
Lymphocyte, ×10^9^/L	1.62 ± 0.66	1.62 ± 0.64	0.973
Platelets, ×10^9^/L	193.04 ± 64.55	200.34 ± 66.84	0.6
AST, U/L	93.48 ± 124.31	87.77 ± 110.63	0.819
ALT, U/L	47.96 ± 52.01	47.59 ± 39.62	0.970
GGT, U/L	181.35 ± 253.85	271.34 ± 375.69	0.185
TBIL, μmol/L	22.41 ± 18.50	18.08 ± 10.76	0.177
DBIL, μmol/L	8.82 ± 9.57	6.76 ± 4.32	0.189
IBIL, μmol/L	11.32 ± 7.39	10.68 ± 6.50	0.699
Total cholesterol, mmol/L	4.99 ± 1.46	5.42 ± 3.84	0.516
HDL, mmol/L	1.69 ± 0.64	1.55 ± 0.85	0.403
LDL, mmol/L	2.36 ± 1.06	2.29 ± 0.81	0.76
Triglycerides, mmol/L	1.71 ± 1.38	3.20 ± 5.43	0.105
Serum creatinine, μmol/L	60.55 ± 17.95	61.17 ± 18.18	0.871
UA, μmol/L	397.70 ± 160.75	378.27 ± 126.53	0.527
BUN, mmol/L	4.58 ± 2.29	4.23 ± 2.11	0.454
Fasting glucose, mmol/L	5.84 ± 2.18	6.06 ± 3.06	0.702
CK, U/L	377.19 ± 436.60	558.10 ± 1568.33	0.453
CK‐MB, U/L	15.71 ± 7.72	17.59 ± 17.87	0.525
C‐reactive protein, mg/L	5.57 ± 7.65	13.57 ± 32.78	0.157
IL‐6, pg/mL	41.00 ± 9.24	44.44 ± 9.87	0.091
SERPINA3	2464.80 ± 796.89	2958.34 ± 769.72	**0.004** *

* indicates a statistically significant difference.

**TABLE 3:** Binary logistic regression models for ELANE.

	B	SE	Sig.	OR	95% CI	
					Lower	Upper
SERPINA3	0.001	0.000	0.007	1.001	1.000	1.001
Age	0.002	0.024	0.920	1.002	0.957	1.050
Occupation	−0.562	0.812	0.489	0.570	0.116	2.799
Marital status	0.240	0.905	0.791	1.271	0.216	7.486
Constant	−2.037	2.111	0.931	0.130		

Abbreviations: CI, confidence ratio; OR, odds ratio; SE, standard error.

### 4.2 Plasma ELANE as a Predictor of Relapse

**FIGURE 2:**
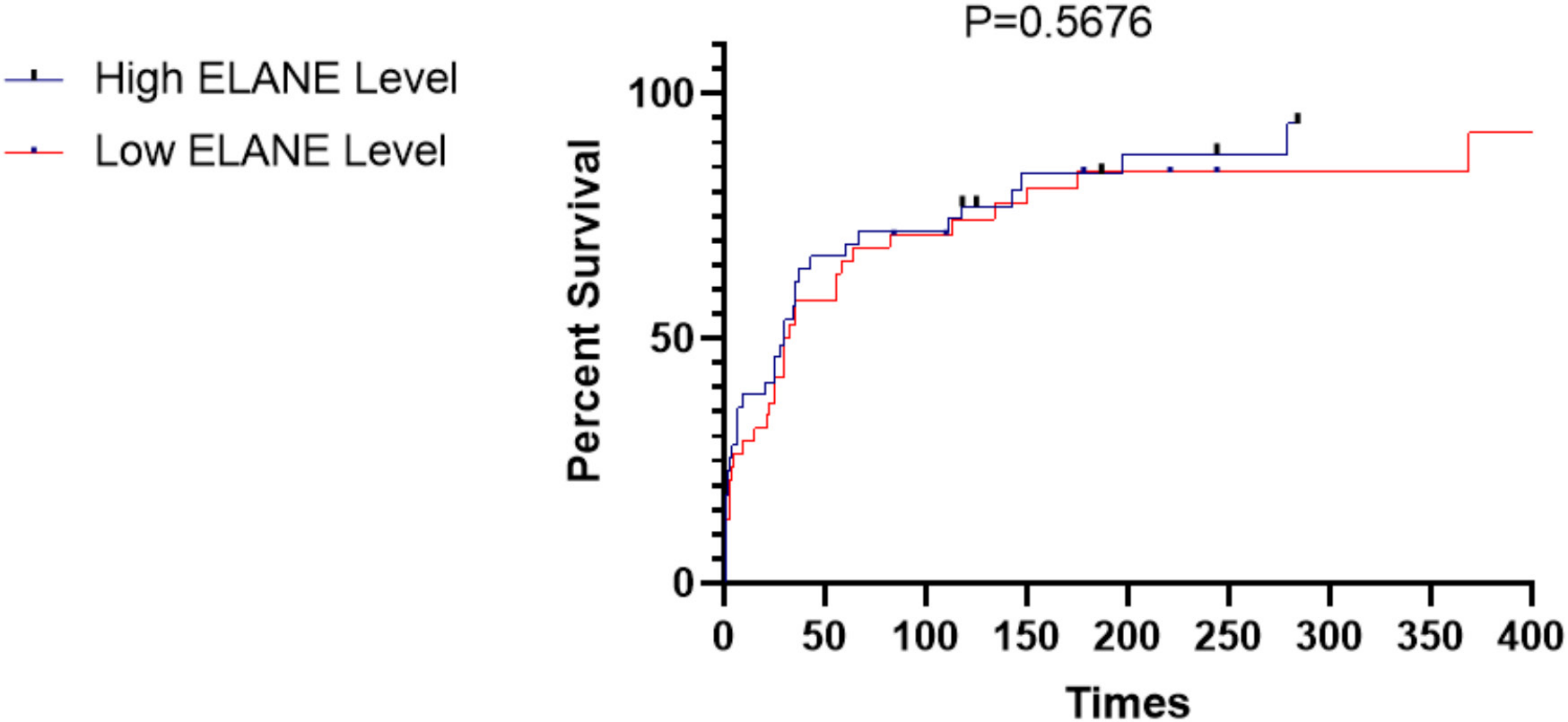
Kaplan–Meier curves based on ELANE levels in patients with alcohol use disorder during follow‐up of up to 12 months (*p* = 0.5676).

**FIGURE 3:**
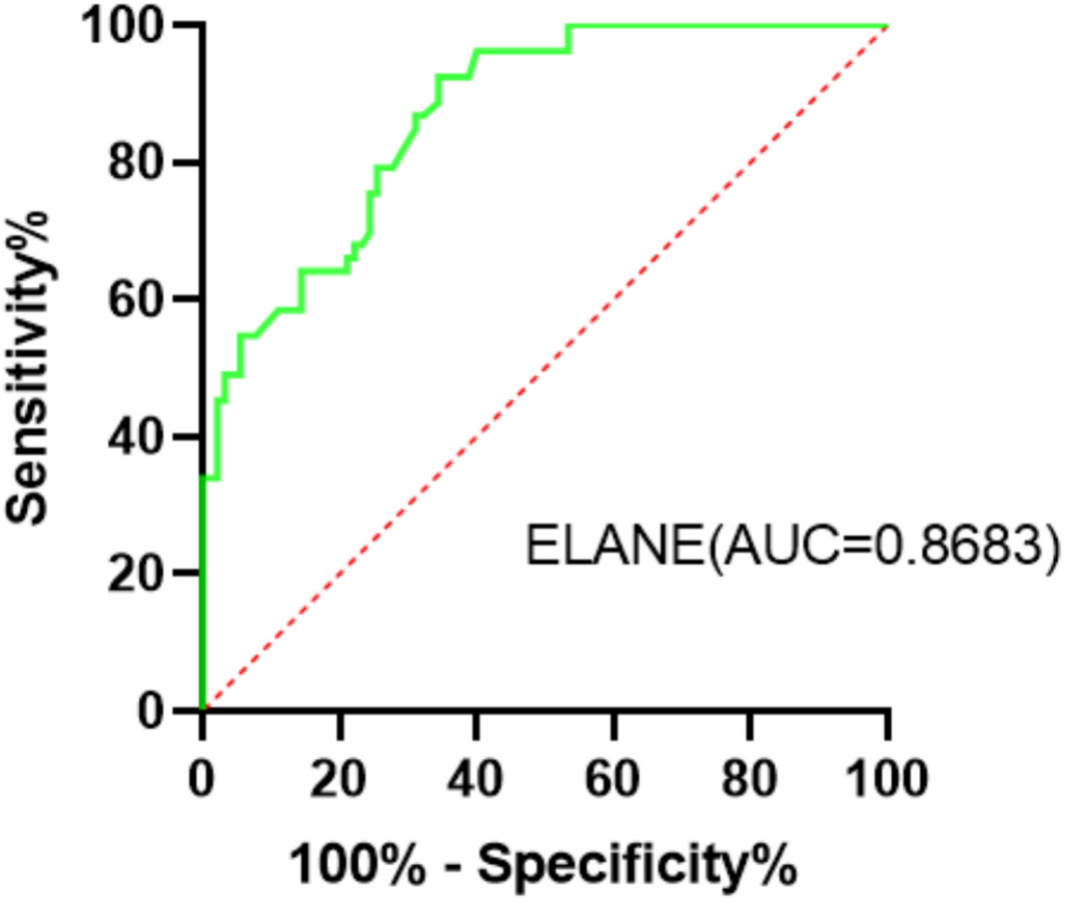
Receiver operator characteristic (ROC) curve based on ELANE levels in patients with alcohol use disorder. The area under the curve (AUC) for ELANE was 0.8683 (*p* < 0.0001), sensitivity was 65.6% and specificity was 92.5%.

At 1 year follow‐up, Kaplan–Meier survival analysis did not show any difference in event‐free survival between patients with low and high ELANE levels (*p* = 0.568) (Figure 2). ROC analysis was used to test the diagnostic value of ELANE, wherein it revealed that ELANE had an AUC of 0.8683 (*p* < 0.0001), sensitivity of 65.6%, and specificity of 92.5% (Figure 3). We included the collected social and clinical biological indicators of patients as covariates and conducted univariate and multivariate survival analyses using the Cox proportional hazards regression model. Cox regression analysis showed that marital status level was a negative predictor of relapse (hazard ratio = 0.516, *p* = 0.038) (Table 4).

**TABLE 4:** Cox regression analyses for predictors of relapse.

Variable	Univariate analysis		Multivariate analysis	
	HR (95% CI)	*p*	HR (95% CI)	*p*
Marital status	0.470	0.03	0.516	0.038
PLT	1.004	0.037	1.003	0.087

Abbreviation: PLT, blood platelet.

## 5 Discussion

We are the first to demonstrate that plasma ELANE is involved in the pathogenesis of alcohol use disorder. This study showed that the concentration of ELANE was negatively correlated with the concentration of SERPINA3. The ELANE gene regulates the synthesis of neutrophil elastase, which is secreted by neutrophils to fight infection when the body produces an immune response to fight infection [20]. SERPINA3, also known as α‐1‐antichymotrypsin, is an antiproteinase that helps protect tissues from damage during neutrophil exudation and phagocytosis [17]. This protease–antiprotease imbalance has been implicated in various inflammatory diseases and represents a hallmark of dysregulated inflammatory homeostasis [21]. The plasma concentration of SERPINA3 protein increases with prolonged inflammation and is associated with multiple organ damage [22]; this also explains the negative correlation between ELANE and SERPINA3. Neutrophil elastase (ELANE) is a potent anti‐cancer protein that kills cancer cells with multiple genes [23].

AUD is a chronic recurrent disease, often involving multi‐cycle treatment, withdrawal and relapse [24]. Family responsibilities and professional ethics are usually lost in severe cases, resulting in a serious social burden. Therefore, it is urgent to identify the different relapse factors [25]. This study shows that marital status is the leading risk factor for relapse. During the in‐patient interview, we found that patients with a good relationship between husband and wife and a high degree of family support were more confident and determined to quit drinking, suggesting that a good marriage helps patients to successfully quit drinking and improve their quality of life, which is consistent with previous research results [26], emphasizing the crucial importance of the social support system in the rehabilitation process. This finding is strengthened by recent 2024 longitudinal research demonstrating that married individuals exhibit substantially lower alcohol‐related mortality rates, with marriage providing particularly strong protective effects during periods of societal stress such as the COVID‐19 pandemic [27]. In future research, we plan to further investigate the broader dimensions of social support beyond marital status, in order to better understand its potential influence on disease progression and recovery.

This study showed no significant difference in baseline ELANE levels in patients with alcohol use disorder between patients who relapsed and those who did not, suggesting that ELANE is not a risk factor for predicting relapse in alcohol use disorder. Despite their diagnostic utility, it highlights the multifactorial nature of AUD recovery. This observation aligns with contemporary understanding that relapse involves complex interactions between neurobiological vulnerability, psychological factors and environmental stressors [28].

## 6 Conclusion

In conclusion, our study provides the first clinical validation demonstrating a significant association between elevated plasma ELANE levels and alcohol use disorder, underscoring its potential role in AUD pathophysiology. Plasma ELANE serves as a highly specific (AUC = 0.8683) neuroinflammatory biomarker for AUD diagnosis. The observed protease–antiprotease imbalance (ELANE ↑, SERPINA3 ↓) warrants further mechanistic studies to establish a theoretical basis for novel diagnostic and therapeutic strategies, such as neutrophil elastase inhibitors. The main limitation of this study is the small cohort size and the lack of repeated blood sampling during follow‐up, which prevented assessment of longitudinal changes in ELANE and other plasma biomarkers in relation to psychosocial and clinical factors.

## Author Contributions

Bo Zhang designed the study. Jin Ling Zhang provided a literature review and assisted in completing the first draft. Meng Ya Zhu and Yong Xue collected the data. Bo Zhang performed the statistical analysis and wrote the manuscript. Yong Xue and Fan Yong revised the manuscript. Yong Xue and Fan Yong provided financial support.

## Funding

The author has nothing to report.

## Ethics Statement

All participants provided written informed consent. The study was authorized and approved by the Third People's Hospital Ethics Committee of Huai'an and carried out in compliance with the Declaration of Helsinki (IRB number: 2021‐07).

## Conflicts of Interest

The authors declare no conflicts of interest.

## Data Availability

The original contributions presented in the study are included in the article, further inquiries can be directed to the corresponding author.
